# Experimental Evaluation of Airlift Performance for Vertical Pumping of Water in Underground Mines

**DOI:** 10.1007/s10230-021-00807-w

**Published:** 2021-08-14

**Authors:** Parviz Enany, Oleksandr Shevchenko, Carsten Drebenstedt

**Affiliations:** Faculty of Mining and Special Civil Engineering, Technische Universität Bergakademie Freiberg, Gustav-Zeuner Straße 1A, 09599 Freiberg, Germany

**Keywords:** Drainage, Air–water two-phase flow, Efficiency, Submergence ratio

## Abstract

This paper presents experimental studies on the optimization of air–water flow in an airlift pump. Airlift pumps use compressed gas to verticall transport liquids and slurries. Due to the lack of theoretical equations for designing and predicting flow regimes, experimental investigations must be carried out to find the best condition to operate an airlift pump at high efficiency. We used a new air injection system and different submergence ratios to evaluate the output of a simple pump for vertical displacement of water in an underground mine. The tests were carried out in a new device with 5.64 m height and 10.2 cm circular riser pipe. Three air-jacket pipes, at different gas flows in the range of 0.002–0.09 m^3^/s were investigated with eight submergence ratios. It was found that with the same air flow rate, the most efficient flow of water was achieved when an air jacket with 3 mm diameter holes was used with a submergence ratio between 0.6 and 0.75. In addition, a comparison of practical results with two theoretical models proposed by other investigators showed that neither was able to accurately predict airlift performance in air–water flow mode.

**Supplementary Information:**

The online version contains supplementary material available at 10.1007/s10230-021-00807-w.

## Abstract (de)

In diesem Artikel werden experimentelle Untersuchungen zur Optimierung der Zweiphasenströmung in einer Mammutpumpe vorgestellt. Mammutpumpen verwenden komprimiertes Gas, um Flüssigkeiten und Schlämme zu heben. Da es keine theoretischen Gleichungen für die Auslegung und Vorhersage von Strömungsregimen in Mammutpumpen gibt, müssen experimentelle Untersuchungen durchgeführt werden, um einen effizienten Betrieb einer Mammutpumpe zu ermitteln. Zur Bewertung der Leistung einer Mammutpumpe in einem Bergwerk wurden ein neues Lufteinspritzsystem und verschiedene Eintauchverhältnisse getestet. Die Versuche wurden an einem Gerät mit 5,64 m Höhe und einem Steigrohr mit 10,2 cm Durchmesser durchgeführt. Dabei fanden drei Luftmantelrohre mit jeweils acht unterschiedlichen Eintauchverhältnissen bei Luftströmen im Bereich von 0,002 bis 0,090 m^3^/s Verwendung. Der effizienteste Wasserdurchfluss wurde bei gleichbleibendem Luftdurchsatz mit einem Luftmantelrohr mit Löchern von 3 mm Durchmesser bei einem Eintauchverhältnis von 0,60 bis 0,75 erreicht. Darüber hinaus wurden die Ergebnisse mit zwei theoretischen Modellen verglichen, die von anderen Forschern vorgeschlagen wurden. Die beiden Modelle waren nicht in der Lage, die Lufthebeleistung in der Zweiphasenströmung genau vorherzusagen.

## 抽象的

介绍了气举泵气-水流量优化的实验研究。气举泵利用压缩气体垂向输送液体和浆液。由于缺乏设计和预测流动机制的理论方程, 需采用实验方法确定气举泵高效运行的最佳条件。用新的空气注入系统和不同的浸没比评价了井下单泵垂向泵水量。测试在高度5.64 m和10.2 cm圆形立管的新装置中进行。用8个浸没比试验了气流量范围0.002–0.09 m^3^/s的三个空气夹套管 (air-jacket pipe) 。结果发现, 在空气流量相同时, 空气夹套管 (air-jacket pipe) 气孔直径为3mm和浸没比在0.6到0.75之间的水流效率最高。另外, 实验结果与其他研究者提出的两个理论模型对比, 显示这两个模型都不能准确预测气-水流动模式下的气举性能.

## Resumen

Este artículo presenta estudios experimentales sobre la optimización del flujo de aire-agua en una bomba airlift. Las bombas airlift utilizan gas comprimido para transportar verticalmente líquidos y lodos. Debido a la falta de ecuaciones teóricas para el diseño y la predicción de los regímenes de flujo, es necesario realizar investigaciones experimentales para encontrar las mejores condiciones de funcionamiento de una bomba airlift con alta eficiencia. Utilizamos un nuevo sistema de inyección de aire y diferentes relaciones de inmersión para evaluar el rendimiento de una bomba sencilla para el desplazamiento vertical de agua en una mina subterránea. Las pruebas se realizaron en un nuevo dispositivo de 5,64 m de altura y con un tubo ascendente circular de 10,2 cm. Se investigaron tres tuberías con camisa de aire, con diferentes caudales de gas en el rango de 0,002–0,09 m3/s y con ocho relaciones de inmersión. Se comprobó que, con el mismo caudal de aire, el flujo de agua más eficaz se conseguía cuando se utilizaba una camisa de aire con orificios de 3 mm de diámetro con una relación de inmersión entre 0,6 y 0,75. Además, la comparación de los resultados prácticos con dos modelos teóricos propuestos por otros investigadores demostró que ninguno de ellos era capaz de predecir con exactitud el rendimiento del transporte aéreo en el modo de flujo aire-agua.

## Introduction

An airlift pump is a device for transporting slurries and liquids using compressed gas injection. More than a two hundred years ago, Carl Luster introduced the concept of the first airlift pump in Germany (Ahmed et al. 2016). The pump consists of a vertical riser pipe and an air injector; the injector is often installed at or near the bottom of the riser pipe. The action of the pump is based on changing the density of the liquid inside the riser pipe by injecting compressed air (Abed et al. 2018). Then the drag force between the formed air bubbles and the water helps the liquid phase to move upwards (Pougatch and Salcudean 2008). The most common examples reported in the literature for using this pump are sewage treatment plants (Kalenik 2015), raising liquid hydrocarbons in the oil industry (Clark and Dabolt 1986), shaft and well drilling (Maliky 2014), and mining of minerals from the ocean (Khalil et al. 1999). This type of pump is especially appropriate for situations where other pumping methods face technical problems such as: when the water is corrosive (due to the high cost of repairs for conventional pumps), where it is not possible to filter water and the ability to simultaneous transfer sediment and water vertically is required, where pre-treatment of water by blowing in some useful gas is needed, such as for mitigation of acid mine drainage (AMD), and for pumping sparkling water, especially if the percentage of gas in the water exceeds 14% (Awari et al. 2004).

Despite the simple structure of the airlift system, there is no proper theoretical model for designing the different parts of it (Ligus et al. 2019; Samaras et al. 2005) because the details of the flow characteristics have not yet been accurately determined. Hence, creating optimal economic conditions for the operational performance of the airlift pump based on the latest theoretical research is difficult, due to the method’s broad diversity and varied dimensions.

Many researchers (Cacharda and Delhaye 1996; Clark and Dabolt 1986; Lockhart and Martinelli 1949; Nicklin 1963; Stenning and Martin 1968) have tried to offer possible formulae based on a variety of theories such as first law of thermodynamics, adiabatic air–water flow, two phase slug flow, and one-dimensional flow. Unfortunately, the formulae have not yet achieved significant success in predicting the performance of this device under different conditions. As an example, Jeelani et al. (1979) showed that the theoretical equations presented by Hussain and Spedding (1976) are not applicable to riser pipes with diameters larger than 8 mm.

In addition, for convenience in calculations and easier expression, some researchers have ignored parameters in their theoretical equations, which reduced the precision of their formulae. Nicklin (1963) presented a theoretical formulation for the performance of an airlift with air–water flow but neglected the entrance effects of air and water in the riser pipe and assumed that air–water flow in the riser tube was in slug form. Sharma and Sachdeva (1976) showed that increasing the inlet air velocity caused the flow of air bubbles inside the riser tube to change from bubbly to slug flow. This process can continue to occur up to a point where the air–water flow changes to annular flow, which greatly reduces vertical water displacement.

The use of photographic techniques showed that there were actually four different types of air–water flow regimes in the riser tube (Francois et al. 1996; Taitel et al. 1997), depending on the amount of influent air. Therefore, theoretical equations obtained based on only one of the flow regimes can certainly not predict the overall performance of the pump in different conditions.

Researchers have tested different ideas to improve the pumping range and efficiency of this device. These experiments led to the discovery of which factors can most affect the performance of an airlift, and so these should be incorporated into the theoretical equations. Among them, the most important parameters are gas bubble dimension, the changing density and momentum of the air–water mixture in the air jacket (a perforated tube for radial air injection into a riser tube), the friction of air with the inner tube wall, and the temperature of water. The latter has less effect than the others (Oueslati et al. 2017).

The role of gas bubbles can be examined from various aspects but principally the aspects fall into two groups: smaller bubble size and delay of bubble agglomeration. Parker (1980) investigated the effect of two models of air injectors in air–water flow. He found that a combination of high air flow rates with small holes in the footpiece (a perforated disk at the base inlet of the riser pipe for axial air injection) improved the capability of vertical water transportation with airlift. Awari et al. (2004) showed that increasing the diameter of a tapered riser tube delayed air bubble accumulation and improved the efficiency of water pumping from great depths.

To reduce the friction of flow with the inner riser tube wall, Ahmed and his colleagues (2016) designed a novel type of air jacket that could inject compressed air radially and axially close to the riser tube wall. In 1979, Khalil and Elshorbagy also reduced the friction coefficient by designing a new riser tube that raised the efficiency of the pump in air–water flow. In addition, to decrease the friction and lower pressure loss in the pipe, a drag reduction agent was introduced to aid the air–water flow. This component loses efficiency over time by mechanical and thermal effects. Therefore, surfactant can be used to overcome this problem and also lower surface tension. Based on the four types of air–water flow regimes, the positive effect of this flow improvement has been reported in different ranges, sometimes reaching up to 80% (Liu et al. 2014).

Oueslati and Megriche (2017) investigated the effect of water temperature on the performance of an airlift pump. They increased the water temperature up to 70 °C and at each step it became clear that the pump efficiency increased slightly as the water temperature increased. Since the process of compressing the air increases the temperature of the injected air, it is sometimes necessary to pay attention to the temperature of the air–water mixture to obtain proper performance.

Despite the various researchers that have investigated airlift pumping, there was no adequate study of the outcomes of using this device for mine dewatering. One report by Shaw (1920) describes its use in an underground mine in Mexico but unfortunately, there are insufficient details about the device and its operation (Clark 1986; Francois 1996). In this paper, we provide the results of a practical experiment, focused on improving airlift performance by air bubble control with three different air jackets. Our main objective was to evaluate the conditions of using an airlift for drainage systems in underground mines. Therefore, air–water flow experiments were performed to study the efficient operation of airlift pumping by air injection with eight submergence ratios (the ratio between the immersed length of the riser and its total length) at different air flow rates.

### Experimental Data

The airlift pump was designed at the Technical University Bergakademie Freiberg, with support from the HydroCoal Plus project, funded by the Research Fund for Coal and Steel (European Commission). The pump was installed in the Reiche Zeche experimental mine (Freiberg, Germany) at a depth of 100 m. The tests were carried out in ambient conditions at a temperature of 17.0 °C and static pressure of 97.8 kPa.

**Fig. 1:**
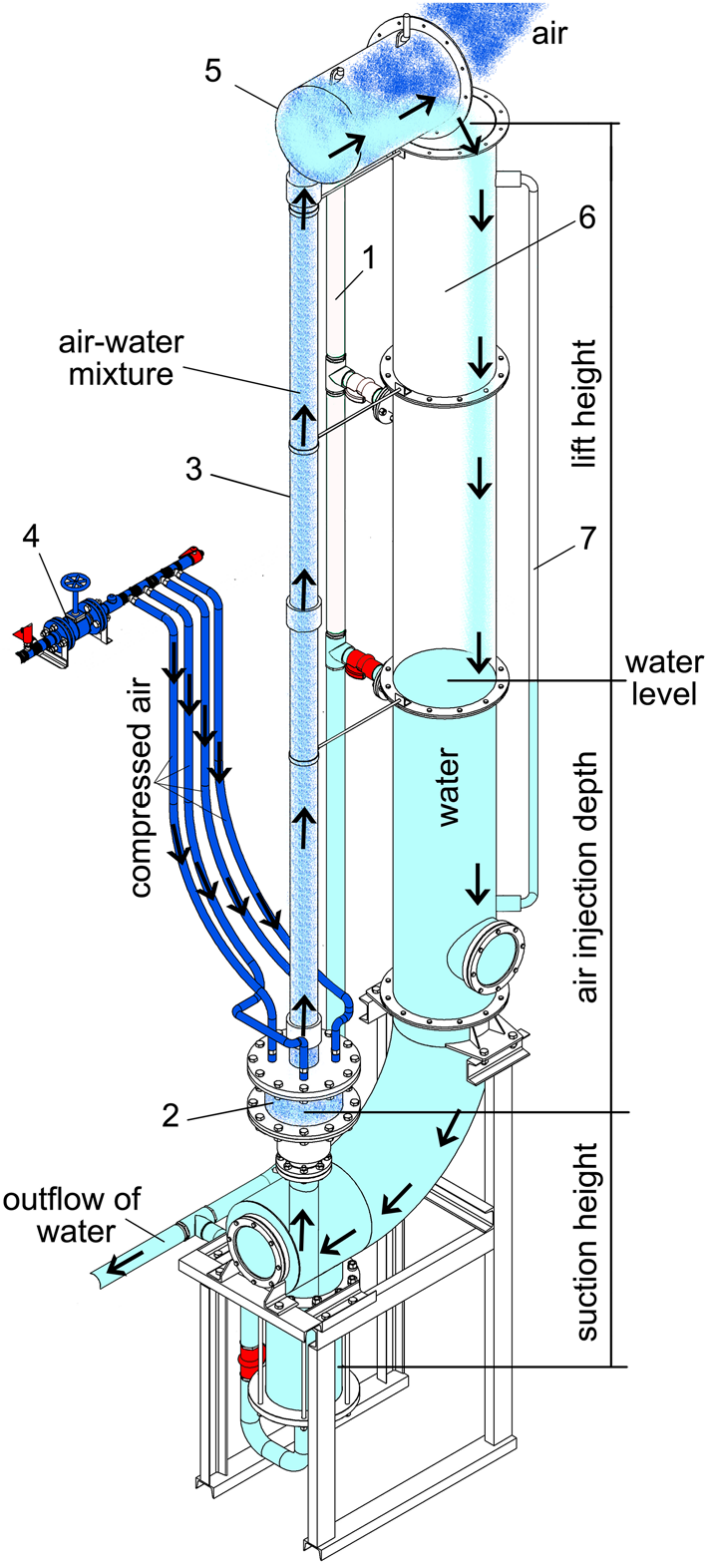
Schematic of working principle of the airlift pump

A model airlift pump is illustrated in Fig. 1. The riser pipe (3) is a transparent tube with internal diameter 10.2 cm and outside diameter 11 cm. The total length of the riser, which is calculated without considering the length of the suction pipe and the air jacket, is 4.26 m. To save water consumption and the possibility of testing the pump at submergence ratios close to one, the airlift is designed to work in closed cycle. That is why the upper part of the riser pipe is connected to one separator tank (5), where the air can be easily separated from the pumped air and water mixture. In addition, the water storage pipe (downcomer) (6) is a steel cylinder with an inner diameter of 41 cm is used to prevent static water level fluctuations.

A rotary screw compressor (Kaeser CS121) was used to supply the compressed air required by the airlift. The compressor has a power of 75 Kw and produced 11.6 m^3^/min of compressed air at a maximum pressure of 8 bar. To prevent air pressure fluctuations during the test, compressed air was first stored in the reservoir and then travelled through a 38.9 mm diameter pipeline to an on/off valve, then to a distributor, where the air is divided into four injection pipes to the air jacket. These four air-supply lines cause the air to be evenly distributed around the air jacket to balance the radial momentum of the air from each side. The air jacket is a cylindrical stainless-steel pipe with circular holes of varying numbers and diameters uniformly distributed around it. Supplemental Figure S-1 shows the schematic structure of the tested air jacket. To equalize the conditions of each injector, the total area of the holes on each injector was chosen equal to 5.9 $\times$ 10^–4^ m^2^ (details are given in Table 1).

**Table 1:** Parameters of investigated air-jacket

Type of air-jacket	Diameter of holes d (mm)	Number of holes
P_1_	3	84
P_2_	6	21
P_3_	9	9

To measure air velocity and pressure, two sensors were installed before the on/off valve: a flow sensor (IFM SA5004) with a measuring range of 2 to 100 m/s and an accuracy of ± 7% measured value + 2% of the final value of the measuring range, and a pressure transmitter (IFM PN3093) with a measuring range from 0 to 25 bar with an uncertainty of less than ± 0.5%. The water flow was measured with a magnetic inductor device from Optiflux with a precision of ≈ 0.5% where it was installed below the air jacket. The air and water temperatures were also measured during the tests. At the beginning of the experiment, the air temperature was constant and its value diminished with time during the pumping from 15 °C to 12 °C. Eight submergence ratios were used to cover the entire experimental range: 0.31, 0.4, 0.5, 0.6, 0.7, 0.75, 0.8, and 0.89; this was controlled by the drainage valve (1) where it connected to the water storage pipe (6) ‒ the numbers refer to locations on Fig. 1. As the air–water mixture began to go up the riser tube, a barometer (7) showed a slight decrease in the static water level. This drop, just like that reported by Maliky (2014), was very small and did not affect the results. To induce circulation of the liquid in the riser pipe, the minimum air velocity was varied at each submergence ratio and measurements began after the airlift operation stabilized. For each intended submergence ratio, the test was performed four times with 5 min intervals between repeated tests within a measuring series. The software collected data at a frequency of two Hz for 500 s.

## Results and Discussion

The experiment was done with three variable parameters: submergence ratio α, air flow rate Q_a_, and type of air jacket (P_1_, P_2_, and P_3_). The main objective of this experiment was to find out the optimal airlift pump configuration for air–water mixture for use in draining an underground mine.

The effect of the compressed air on the air jacket has been described in detail previously (Ahmed et al. 2016; Kalenik 2015; Khalil et al. 1999; Neto et al. 2008; Parker 1980). In this experiment, compressed air was blown parallel to the outer wall of the air jacket and did not hit it directly. This method avoids reducing the initial energy of the compressed air, allows the use all of the holes embedded in the air jacket, and creates an even entry of air into the riser pipe. Our experiment was designed to properly evaluate this new method of parallel air injection and to confirm the influential parameters identified by previous researchers, such as submergence ratio and air flow rate.

Each experiment started at the minimum air velocity required to initiate water circulation in the airlift. In terms of air consumption, the first type of air jacket with the smallest diameter hole required the least amount of air to raise the water to a height of 1 m (see supplemental Table S-1). In each air jacket, if the amount of injected air was less than the minimum, the gas bubbles could not provide the buoyant force required to move water upwards to pass through the water column and toward the separator tank (5).

Especially for submergence ratios less than 0.6 and low air flow rates, water flow fluctuations were visible in the riser pipe. Therefore, the mixture of water and air moved upwards irregularly. The flow fluctuation was such that the mixture of water and air fell slightly in the opposite direction of the main flow and continued to move vertically when the next high-pressure flow arrived. This phenomenon is considered an obstacle to the proper operation of the pump in the case of air–water flow. The water can move upwards more regularly (less flow fluctuations) if the amount of incoming air or the submergence ratio is increased. It is worth noting that a small amount of the flow irregularity in low submergence ratio pumping is due to water falling from the separator tank into the water storage pipe. This influence of a submergence ratio less than 0.6 can be seen clearly by the difference between the static pressure level and dynamic pressure in the barometer (7), which was installed along the outside of water storage pipe (downcomer).

Water flow fluctuations in the riser pipe also depends on hole dimensions in the air jacket. Compared to the other air jackets, type P_1_ produced very small air bubbles in the entire cross-section of the riser pipe, which decreased the amount of oscillation, caused the upward flow to be more regular form, and improved the pump’s effectiveness. A similar phenomenon was also reported by Kalenik (2015).

As previously mentioned, the performance of the pump depends on the flow rate and pressure of the incoming air, the submergence ratio, and the friction of the fluid with the pipe wall. Due to the similarity of the friction in all of the experiments, this factor did not play an important role in this research, so the arithmetic average values measured for the other effective parameters were used.

**Fig. 2:**
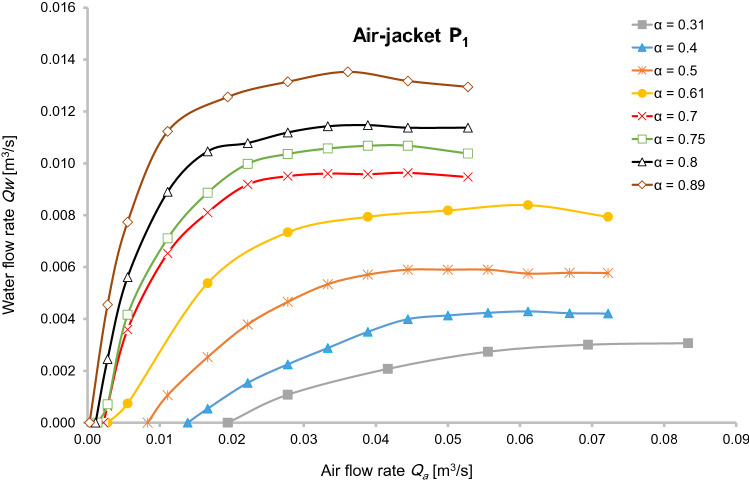
Comparison of water flow rates with different submergence ratio for air-jacket P_1_

The effect of incoming air on the velocity of the transported water for different submergence ratios is presented in Fig. 2 for mixer P_1_. The starting point of each graph corresponds to the amount of air required for vertical water transfer up to 1 m from static level. Due to the short distance between the water in the riser pipe to the separator tank, this rule did not apply to submergence ratios of 0.8 and 0.89. Therefore, the beginning of water circulation in the airlift was considered the starting point of the graph for those two submergence ratios.

Figure 2 reveals the effect of air flow and submergence ratio on water flow rate in air jacket P_1_. For submergence ratios exceeding 0.5, the water flow increased with a steeper slope, and after reaching the maximum, the effect of further air injection was insignificant. Figure 2 shows that for the same amount of air, the water flow was largest at a submergence ratio of 0.89. One explanation for this is that the travel distance for an air bubble increases as the submergence ratio increases. Hence, air bubbles have enough time to transfer their energy to the water molecules, causing increased water flow. The velocity of compressed air did not show the same trend as the water transfer rate. For submergence ratios less than *α* = 0.5, much more air is required, e.g. 0.02 m^3^/s for *α* = 0.3, to initiate the transfer of water up the pipe. At higher submergence ratios, the water reaches considerable flow rates, even with just a small amount of compressed air.

In a study on a 4 m high airlift and three different types of air injectors, Kalenik (2015) concluded that the air flow and air pressure contributed more than 95% to the water pump flow, with all other factors contributing only 5%. If we consider the best results of the present practical experiment (Fig. 2), it can be seen that increasing the air velocity up to 0.03 m^3^/s at a submergence ratio of 0.89 enhanced the displaced water by up to 67%, while the same air flow and a submergence ratio of *α* = 0.6 increased the ultimate yield by 40%. Therefore, Kalenik (2015) ignored the effect of other parameters such as submergence ratio. Raising the air velocity will be important whenever we can transfer the energy of the compressed air to the water in an optimal way, but will not always be the most effective in improving water pumping.

**Fig. 3:**
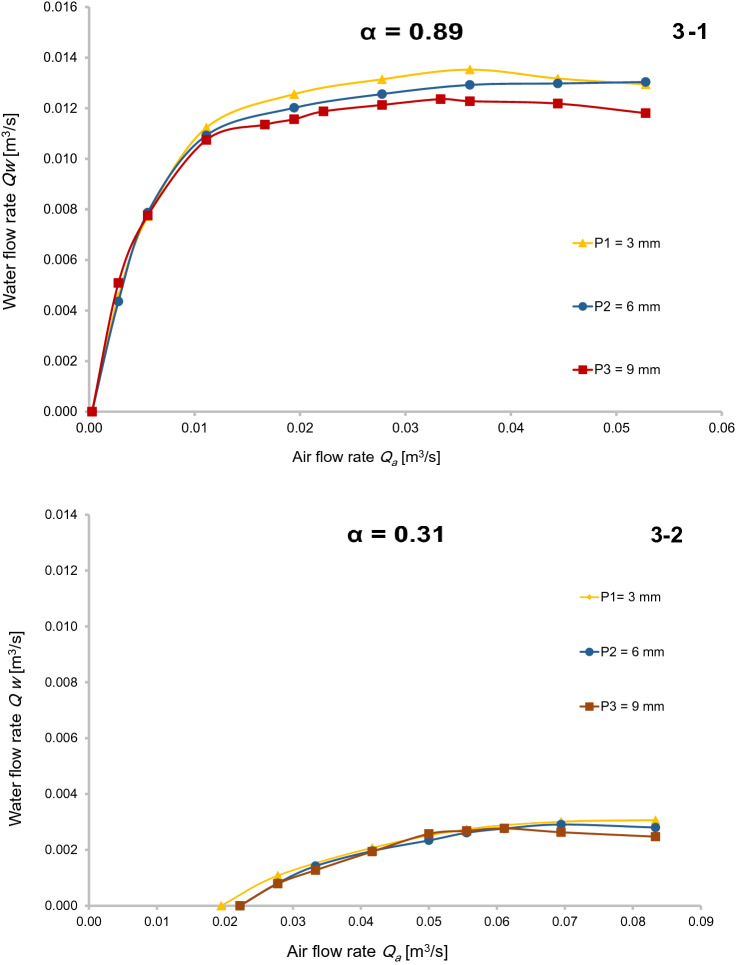
Comparison of water flow rate with different air-jackets for *α* = 0.31 and 0.89

As can be seen from Fig. 3(1), increasing the volume of influent air kept increasing the water flow rate until it reached a maximum value. After the peak water flow was reached, increasing the influent air no longer affected the water flow rate. Instead, the flow rate was fairly constant and even show eventual slight reductions.

Relative to the mass flow of water created by a given injected air mass flow, with a submergence ratio of 0.89, all of the experimental data for each air jacket tended to produce similar flow rates, as can be seen from the three curves in Fig. 3(1), especially for low values of air flow (less than 0.012). Similar conditions are observed for the submergence ratio of 0.31 in Fig. 3(2). In general, the flow of water increases slightly for air jacket holes when the submergence ratio is constant. Parker (1980), in his research on a small airlift with radial air injection conditions, found that the performance of the device was not affected by the number and diameter of holes in the air jacket. Parker’s result cannot be generalized to all devices since Parker considered only one submergence ratio (*α* = 0.55). It can be inferred from Fig. 3(1, 2), under certain conditions of air flow and submergence ratio, that air jacket P_1_, with smaller and more drilled holes, had a greater effect on the pumping efficiency compared to the other mixers, as long as the compressed air was injected parallel into the manifold.

### Comparison with the Theoretical Formulas

Here we compare the results of our applied research with the two analytical studies presented by Stenning and Martin (1968) and Hussain and Spedding (1976) to predict the discharge of water. The Stenning and Martin equation was obtained by a combination of theoretical and practical research and assuming one-dimensional flow in the airlift and establishing momentum continuity between the inlet and outlet of a lifting tube. Due to the lack of sufficient explanation to use this formula, researchers have been proposed different methods. As inferred from Parker (1980), with an initial guess for S (slip ratio) and K (friction factor), the results of calculations should be plotted as two dimensionless parameters $\frac{V}{\sqrt{2gL}}$ and $\frac{{Q}_{g}}{{Q}_{f}}$, where V is the velocity of the water in the entrance of the suction pipe, g is gravitational acceleration, L is the length of the riser pipe, Q_g_ is the air flow rate, and Q_f_ is the water flow rate.

**Fig. 4:**
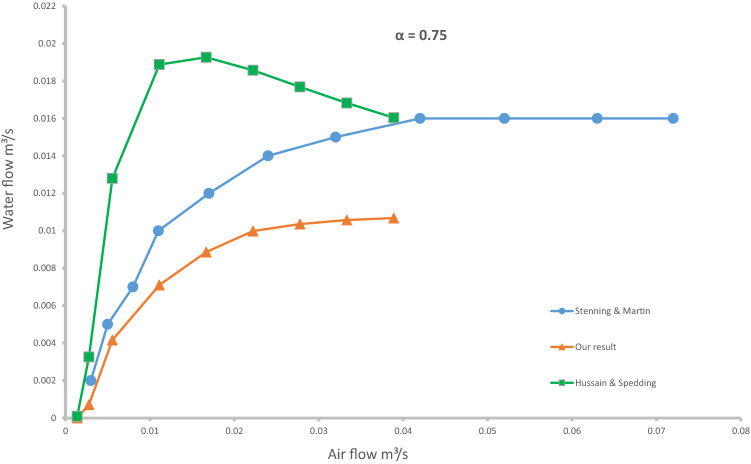
Comparisons between the theoretical model and experimental results

Meanwhile, Kassab et al. (2009) suggested an iterative procedure. First, we have to assume a value for the water flow rate and use it to calculate parameters S and K. Then the value obtained from the calculations for the water flow rate should be compared with the initial guess; if the difference is less than 0.001, then the calculations should be stopped, otherwise the steps should be started again from the beginning. Both procedures were tested to calculate the outflow of water, but the final results of calculations were clearly different. Since our experiments were performed on an airlift with a riser tube diameter of 10.2 cm, Stenning and Martin also provided a prediction of performance for an airlift with a same diameter. The only difference was the length of riser pipe, which was about 6.5 m longer than our device. The results of that prediction are plotted against our experimental data for a submergence ratio of 0.75 in Fig. 4, which shows that Stenning & Martin’s methods underestimated the amount of air required to start the vertical water transfer. This inference is due to the fact that Weber (1974) in his research on pipes with different lengths and same diameter showed that, as the length of riser pipe increased, more air was needed to achieve the same water flow rate than when the pipe was shorter. Therefore, the starting point of the graph should be located further right of our test data. Parker (1980) also confirmed that the Stenning and Martin model did not accurately predict water flow at low air flow.

The results of water flow estimation by Hussain & Spedding’s formula are also displayed in Fig. 4. It is quite clear that their model overpredicts the amount of water flow. In particular, when the inlet air flow was low, the difference between the calculated values and experimental data was up to four-fold. However, as the amount of incoming air increases, the difference in results decreases. In Hussain and Spedding’s model, the water flow rate goes to maximum quicker than our experimental data as well as the model from Stenning and Martin. Then the water flow suddenly starts to decrease, which is contrary to the usual patterns. Sawiski et al. (1999) also compared Hussain and Spedding’s formula for a small-scale airlift, but he did not find any similarities between theory and experiment. Altogether, Hussain and Spedding’s model is not capable to predicting the full range of water flow for an airlift. The source of this error can be found in two empirical coefficients K_1_ and K_2_, which they estimated from numerical comparisons with other experimental results.

### Efficiency

For each experiment, the theoretical efficiency of the airlift pump can be calculated using the following equation (Reinemann et al. 1990):

$$\eta =\frac{{N}_{2}}{{N}_{1}}$$

**Fig. 5:**
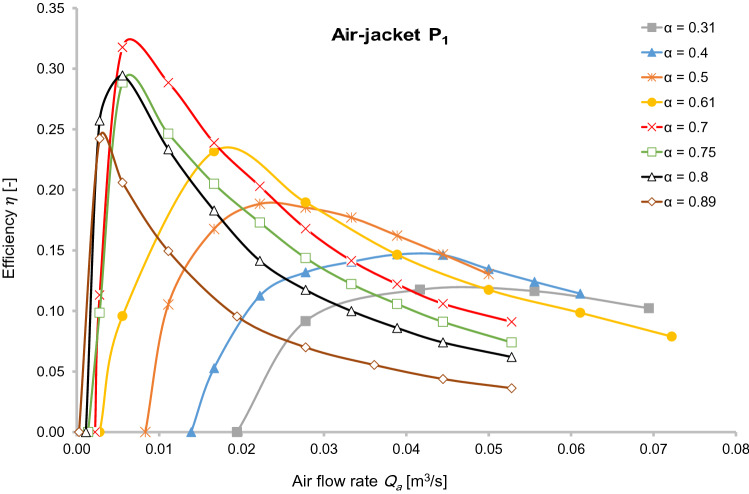
Pump efficiency and air flow rate at different submergence ratio for air-jacket P_1_

This ratio is defined as an energy (N_2_) required to pump the pure water to the separator tank, compared to the energy (N_1_) used by the compressor to achieve isothermal compression of the air from atmospheric pressure to the air injection pressure. The results of this calculation are plotted for mixer P_1_ in Fig. 5.

In Fig. 5, it is apparent that for submergence ratios greater than 0.6, a similar efficiency can be achieved for low airflow values and that the highest efficiency was associated with injecting compressed air at less than 0.01 m^3^/s. Figure 5 clearly shows that the economic performance of the airlift is strongly dependent on the submergence ratio. For a submergence ratio less than 0.6, the efficiency obtained from pumping water is, at best, ≈ 20%, which is possible at high air injection rates. As an example, for α = 0.31 of compressed air injection, a rate of 0.05 m^3^/s yields maximum efficiency.

**Fig. 6:**
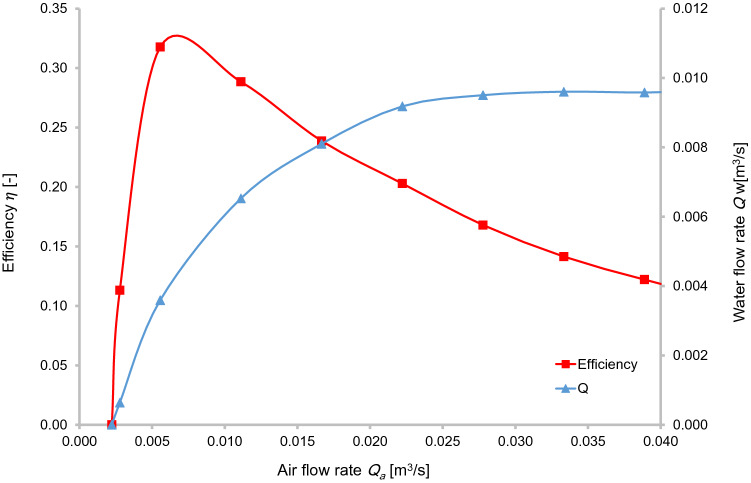
Water flow rate against air flow rate and efficiency for air-jacket P_1_ and *α* = 0.7

Figure 6 shows the correlation between the experimental efficiency and water lifting rate for mixer P_1_ at a submergence ratio of 0.7. It is quite clear that the maximum efficiency of the pump does not belong to the time when we have the maximum rate of displaced water. This result emphasizes that increasing the amount of influent air cannot help much to enhance the airlift efficiency. Therefore, depending on the conditions of the mine, one can choose between the economic efficiency of pumping operation and draining water faster.

There are different opinions about the most effective submergence ratio for the optimum output of the airlift. Tighzert et al. (2013) concluded that the best performance of their 3 m long airlift was achieved at a submergence ratio of 0.75, and that beyond that, the efficiency declines. Their recommended optimal range of submergence ratios for proper device performance was 0.4 to 0.75, which is slightly different from our results. The discrepancy could be due to the dimension of our airlift and the pressure of the compressed air, which were both higher under their test conditions than in our study. We found that the optimum range of submergence ratios was from 0.6 to 0.75. Outside this range, the performance of an airlift for two phase flow drops sharply and would likely be not economical.

**Fig. 7:**
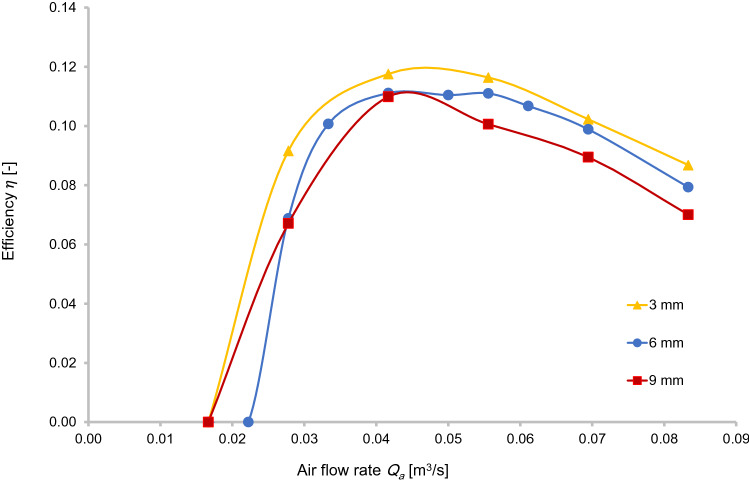
Lifting efficiency for three air-jackets at a submergence ratio of 0.31

Compared to other types of air jackets, the P_1_ type performed the best for vertical water transport with this airlift design (Fig. 7). P_1_ was much as 10% more efficient than types P_2_ and P_3_. The mixer P_1_ produced smaller and more regular air bubbles to enhance the delivery of water in the separator tank by creating more shear stress between the water molecules and the outer surface of air bubbles. Increasing the diameter of the holes in the air-mixer reduces the efficiency of the pump; that is why the performance of P_2_ was better than P_3_. This condition was not always the same due to flow fluctuations and precision of the sensors, but in 90% of the cases, P_2_ provided better efficiency and maximum water flow rate compared to P_3_.

The results of our practical experiments show that to efficiently use an airlift pump for underground mine drainage, it is necessary to have a suitable height of water so that a submergence ratio of 0.6–0.75 can be ensured. This in turn requires proper collection and delivery of water to the pumping site or finding a suitable place for a well in the aquifer. To give an illustration, if the water must be pumped 60 m above its static level, assuming an economic working condition (*α* = 0.75), this requires a well with a minimum water depth of 180 m. Providing such a situation creates its own problem in underground environments.

## Conclusion

A preliminary design of an airlift pump using three type of air jackets and eight different submergence ratios, utilizing parallel injection of the compressed air into the manifold, was investigated experimentally. Based on the test results, the following main conclusions are drawn:

1. One of the effective parameters for airlift performance is to use an air jacket with smaller hole diameters. In terms of air consumption at a constant submergence ratio, the first type of air jacket P_1_ with more of the smallest hole diameters (3 mm) required the least amount of air to start lifting water. Moreover, it was more efficient in pumping water than air jackets with bigger hole diameters (P_2_ and P_3_).
2. Water flow fluctuations in the riser pipe reduce the efficiency of the airlift for vertical water transfer. To prevent this phenomenon, it is necessary to correctly choose the amount of compressed air injection and the submergence ratio.
3. The amount of compressed air needed to have a continuous upward flow and maximum efficiency is different for each submergence ratio. However, the same trend exists for water pumping with an airlift. The water flow rate increases until an ultimate pumping rate is reached, beyond which the liquid flow rate remains constant despite increased air flow and finally decreases slightly.
4. According to the results, the maximum submergence ratio is equal to 0.75, and beyond that, the efficiency of water pumping is reduced again, and only faster pumping rate can be achieved. The optimal submergence ratio for useful efficiency is between 0.6 and 0.75 if the compressed air is injected parallel into the manifold. However, the pumping height of water at submergence ratios above 0.6 is not significant. Thus, the airlift is more economical for pumping water at short altitudes above the static water level.
5. We found that neither of the two theoretical models (Hussain and Spedding 1976; Stenning and Martin 1968) compared well with our experimental results. To use these theoretical models to predict pumping performance with acceptable accuracy, it is necessary to modify them to estimate flow parameters for a wide range of pumping conditions with minimum deviations.

## Supplementary Information

Below is the link to the electronic supplementary material.

Supplementary file1 (TIF 422 KB)

Supplementary file2 (DOCX 1615 KB)
