# Supplementary figures and images for: Experimental Evaluation of Airlift Performance for Vertical Pumping of Water in Underground Mines
Source: Mine Water Environ. 2021 Aug 14;40(4):970–9. doi: 10.1007/s10230-021-00807-w (PMC8645540; doi:10.1007/s10230-021-00807-w)

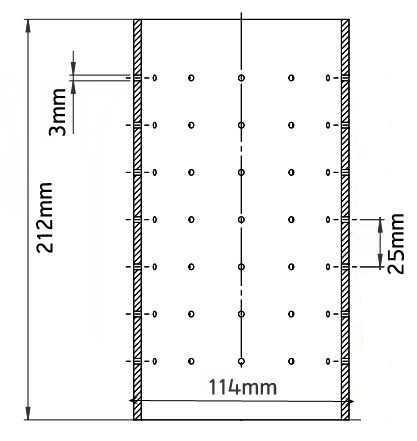

Supplement: Supplementary file 1 — Supplementary file1 (TIF 422 KB) [file 10230_2021_807_MOESM1_ESM.tif]
