# Supplementary material for: Experimental Evaluation of Airlift Performance for Vertical Pumping of Water in Underground Mines
Source: Mine Water Environ. 2021 Aug 14;40(4):970–9. doi: 10.1007/s10230-021-00807-w (PMC8645540; doi:10.1007/s10230-021-00807-w)
Supplement: Supplementary file 2 — Supplementary file2 (DOCX 1615 KB) [file 10230_2021_807_MOESM2_ESM.docx]

**Table S-1** Minimum air required to transfer water up to a height of one meter in the riser pipe for different air-jackets

| Submergence ratio  (α) | Air flow rate for P_1_ (m^3^/s) | Air flow rate for P_2_ (m^3^/s) | Air flow rate for P_3_ (m^3^/s) |
| --- | --- | --- | --- |
| 0.31  0.61  0.7  0.8 | 0.0057  0.0021  0.0014  0.0011 | 0.0078  0.0025  0.0021  0.0017 | 0.0088  0.0034  0.0026  0.002 |

ss
